# Malnutrition and micronutrient deficiencies among elderly persons attending University College Hospital, Ibadan: a pilot study

**DOI:** 10.11604/pamj.2024.48.163.42544

**Published:** 2024-08-08

**Authors:** Temitope Hannah Farombi, Olufisayo Oluyinka Elugbadebo, Oladimeji Adebayo, Joseph Yaria, Lawrence Adebusoye, Temitope Alonge

**Affiliations:** 1 Department of Neurology, University College Hospital, Ibadan, Nigeria,; 2 Department of Psychiatry, University College Hospital, Ibadan, Nigeria,; 3 Institute of Cardiovascular Diseases, University of Ibadan, Ibadan, Nigeria,; 4 Chief Tony Anenih Geriatric Center, University College Hospital, Ibadan, Nigeria,; 5 Department of Orthopedic and Trauma, College of Medicine, University of Ibadan, Ibadan, Nigeria

**Keywords:** Malnutrition, micronutrient, elderly, nutritional assessments, dietary patterns

## Abstract

**Introduction:**

malnutrition and micronutrient deficiencies are pressing health concerns, particularly among the elderly. As this population is vulnerable to nutritional imbalances, understanding the prevalence and contributing factors is crucial for designing targeted interventions. This pilot study focuses on assessing the extent of these issues among elderly people attending a geriatric center in the University College Hospital, Ibadan.

**Methods:**

this study employs a cross-sectional design, involving a sample of elderly individuals attending a geriatric center at the University College Hospital, Ibadan. Anthropometric measurements and dietary assessments were conducted using the Mini Nutritional Assessment (MNA) tool. A structured questionnaire was used to gather information on socio-demographic factors and other medical parameters. Continuous and categorical variables were compared respectively by Student’s t-test or Chi-square test respectively.

**Results:**

in this study, findings indicated that none of the participants exhibited malnutrition. Instead, 72.7% demonstrated a normal nutritional status, while 27.3% were identified as being at risk of malnutrition. A lesser fraction had deficiency of vitamins A (10%) and D (1%). Furthermore, males aged 60-69 years and those above 80 years showed a higher likelihood of a favorable nutritional assessment compared to females.

**Conclusion:**

the study revealed an absence of malnutrition among the elderly individuals attending the geriatric center in the University College Hospital, Ibadan. Notably, females had a higher probability of malnutrition compared to males. These findings underscore the importance of targeted nutritional interventions, especially among at-risk groups, to promote the overall well-being of this population.

## Introduction

Conventionally, elderly people are considered adults who have attained a chronological age of 60 years and above [1]. They are within a population group fraughted with several co-morbid conditions. Globally, a rapid rise in their population is anticipated within the next 30 years [2]. Similarly, in sub-Saharan Africa, Nigeria inclusive, the elderly population is steadily on the rise despite all odds [1]. World Health Organisation (WHO) defines malnutrition as “an imbalance between supply of nutrients and energy and the body´s demand for growth and maintenance of tissue function” [3].

Generally, micronutrient deficiencies across all age groups are considered uncommon in developed countries [4]. However, regardless of economic stage, the elderly are a critical group of people at risk of malnutrition and micronutrient deficiencies [4]. It has actually been demonstrated that malnutrition with micronutrient deficiency is a common problem in both developed and developing countries in their age group [5].

In most African countries, there is dearth of data concerning malnutrition. Micronutrient deficiency has been termed hidden hunger and contributes about 7% to the total world disease burden [6]. The most implicated deficient micronutrients are vitamins A and B (thiamine, riboflavin, niacin, B6, B12), iodine, folate, iron, and zinc. Others include beta carotene, vitamins C, D, and E, copper, selenium, calcium, magnesium etc [7,8].

Some factors that put the elderly at risk for malnutrition and micronutrient deficiencies include low outdoor activity hence inadequate sunlight exposure, reduced financial capacity, the intrinsic properties in the physiology of aging particularly gastrointestinal tract, immune and oral health challenges, drug metabolism pathways of some medications used to treat co-morbid conditions interacting with absorption and bio-availability of these nutrients, etc [9].

The prevalence of malnutrition in the elderly differs among the different populations, this might be as a result of lack of extended research regarding the nutritional deficiencies among the aged population. However, data on the nutritional status of the aged is scarce in SSA and in Nigeria. This is likely due to competing interests as most government policies and international donors on nutrition services focus on infants, young children, and pregnant women. It is therefore imperative that data is gathered to design a nutrition policy program for this growing population.

Therefore, in the study, we assessed the prevalence of malnutrition and burden of micronutrient deficiencies, and the influence of gender on this burden among the elderly population attending a geriatric clinic in Ibadan.

**Objectives:** to assess the prevalence of malnutrition among the elderly population attending a geriatric clinic in Ibadan; to assess the burden of micronutrient deficiencies among the elderly population attending a geriatric clinic in Ibadan; to determine the influence of gender on the burden of micronutrient deficiencies among elderly population attending a geriatric clinic in Ibadan.

## Methods

**Study design:** this is a cross-sectional study among participants aged >60 years from the Oyo State community attending Chief Tony Anenih Geriatric Centre.

**Setting:** the study was conducted at the Chief Tony Anenih Geriatric Centre located at the University College Hospital. The centre is purpose-built to cater for the geriatric patient population.

**Participants:** the participants were consecutively recruited from our elderly patients attending the Chief Tony Anenih Geriatric Centre (CTAGC) located at the University College Hospital and who are also community-dwelling adults. The inclusion criteria were consenting participants aged ≥60 years who are not on admission while those not consenting and are inpatients were excluded.

**Variables:** the participants socio-demographic characteristics were explored; gender, educational status, place of birth, marital status, past occupation, the number of children they have, and the frequency of visits per month. Other variables like who visited the participants the most in a month, supplements used by the participants, the things purchased for the participants, exposure to sunlight, their status on the use of comorbid substances, and being on a special diet were explored. Further variables on the chronic conditions experienced by the participants such as blood pressure, digestive or renal problems, coronary heart disease, dental health, day/night time sleeping hours, and food consumption status among others were assessed using a questionnaire.

**Data sources/measurements:** malnutrition was assessed using the Mini Nutritional Assessment (MNA) tool. The MNA tool is used for identifying the risk of malnutrition in the elderly [10]. The tool was used to assess the nutritional status of the participants; normal nutritional status, at risk of malnutrition, and malnourished. The tool has a scoring system of ‘0´ points as minimum and ‘30´ points as maximum. Participants who score >23.5 points indicate normal nutritional status, 17-23.5 points indicate a risk of malnutrition while <17 points is an indication of malnutrition [10].

This tool comprises of anthropometric and global measurements including information on dietary patterns and individual perception of health amongst the elderly which also includes a decrease in meal intake, weight loss exceeding 3 kg, ability to move, immobilized, psychological stress, neuro-psychological disorders, body mass index (BMI), dependent, taking multiple drugs, having skin sores, daily meal frequency, the total intake of protein meals daily, intake of fruits and vegetables, daily intake of liquids, inability to self-feed, nutritional self-perception, self-perceived health, mid-upper-arm-circumference (MUAC) <21 cm and calf circumference (CC) <31 cm [10]. The BMI was calculated using weight (kg)/height^2^ (m^2^). Weight was measured using a floor scale with the participant in light clothing. Standing height, MUAC, and CC were measured using a plastic tape. Mid-arm circumference (MAC) was measured at the midpoint of the relaxed, non-dominant arm, between the tip of the shoulder and the bony tip of the elbow [10]. Knee height and demi span were measured alongside the BMI as this has been reported to accurately predict undernutrition than BMI [11].

**Frequency of interviews and invasive sampling:** the participants were interviewed once for about 15+/-5 minutes. Blood samples of 6mls were collected and analyzed to determine the micronutrient levels of vitamin A, B12, and D and serum iron levels. A blood concentration of retinol in serum was used to measure the nutritional level of vitamin A; a concentration of less than 0.35 µmol/L was considered to indicate severe vitamin A deficiency. The 25-hydroxy vitamin D test was used to measure vitamin D deficiency, the methylmalonic acid (MMA) test was utilized to assess B12 deficiency, and a serum iron test was performed to determine the serum iron test.

**Study size and technique:** convenient sampling technique was used in this study using a Z score of 1.96 with a 95% confidence interval. A total of 99 participants were recruited.

**Quantitative variables:** the quantitative variables included a number of children, the frequency of visits per month and chronic diseases such as high blood pressure. It also included the anthropometry profile of the participants´, weight, height, BMI, waist circumference, hip circumference waist, hip ratio, mid-arm circumference, calf circumference, knee height, and demi-span measurement.

**Statistical method:** the data was initially recorded on case reporting forms and then transferred to a secure electronic database. Statistical analysis was performed using IBM SPSS Statistics version 23.0 for Windows (Armonk, NY: IBM Corp). The data underwent a normality test. Continuous variables were summarized as means and standard deviations, and comparisons were made using one-way analysis of variance (ANOVA) or Student's t-test. Categorical variables were analyzed in terms of frequencies and percentages, with comparisons made using the Pearson’s chi-square test. A two-sided p-value of less than 0.05 was considered statistically significant for all tests.

The prevalence of malnutrition was assessed using the Mini Nutritional Assessment (MNA) and categorized into three levels: 0-7 (malnutrition), 8-11 (at risk of malnutrition), and 12-14 (normal nutritional status). Descriptive statistics determined the prevalence of malnutrition, and the Chi-square test assessed the association between nutritional status and gender. Micronutrient deficiencies and the influence of gender were summarized using means and standard deviations, with comparisons made using one-way ANOVA or Student's t-test to evaluate the burden of micronutrient deficiencies.

**Ethical considerations:** the study approval was sought from the Oyo State Ministry of Health Ethical Review Board (AD13-479-44509B) and approval was given.

## Results

**Demographic characteristics of the study participants:** the mean age of the participants was 71.9 ± 7.0 years, with the majority being between 70 and 79 years old (46%). Most participants had post-secondary education (55.0%), were retired (77%), and married (49%). A significant portion resided in urban areas (71%), with 35% earning more than ₦61,000 monthly, primarily from their children (57%). Regarding alcohol consumption, 74% had never consumed alcohol, and only 3% currently did. In this cohort, 2% had dysphagia, 45% experienced tooth loss, and 52% used supplements (Table 1). The anthropometric profile indicated a mean weight of 73.7 ± 16.5 kg. Females generally weighed more (74.4 ± 17.2 kg), males were taller (172.1 ± 6.2 cm), and females had wider waistlines (100.6 ± 15.3 cm). Significant differences were found in mean height (<0.001), BMI (0.004), hip circumference (0.006), mid-arm circumference (0.03), calf circumference (0.03), demi-span (<0.001), and gender (Table 2).

**Table 1:** socio-demographic profile of the participants

Variables	All	Male	Female	P-value
Age (mean SD)	71.9 ± 7.0	76.0 ± 8.5	71.2 ± 6.5	0.016**
Age category: young old	39 (39.0)	3 (36)	36 (42.4)	0.089
Middle old	46 (46.0)	7 (46.7)	39 (45.9)	
Very old	14 (14.0)	5 (33.3)	9 (10.6)	
Education status: no formal education	3 (3.0)	0	3 (3.5)	0.791
Primary education	15 (15.0)	2 (13.3)	13 (15.3)	
Secondary education	22 (22.0)	2 (13.3)	20 (23.5)	
Post-secondary/university	55 (55.0)	10 (66.7)	45 (52.9)	
Postgraduate education	5 (5.0)	1 (6.7)	4 (4.7)	
Place of dwelling: urban	71 (71)	14 (93.3)	57 (67.9)	0.057
Semi-urban	24 (24)	0 (0)	24 (28.6)	
Rural	4 (4)	1 (6.7)	3 (3.6)	
Occupation: employed	16 (16)	1 (6.7)	15 (17.9)	0.287
Unemployed can work	4 (4)	0(0)	4 (4.8)	
Unemployed cannot work	2 (2)	1(6.7)	1 (1.2)	
Retired	77 (77)	13(86.7)	64 (76.2)	
Marital status: single	2 (2)	1 (6.7)	1 (1.2)	0.040**
Married	49 (49)	12(80)	37 (43.5)	
Divorced	1 (1)	0 (0)	1 (1.2)	
Separated	4 (4)	0 (0)	4 (4.7)	
Widowed	44 (44)	2(13.3)	42(49.4)	
Monthly income (Naira): <10,000	10 (10)	0 (0)	10 (11.8)	0.348
11,000-20,000	19 (19)	3 (20)	16 (18.8)	
21,000-40,000	18 (18)	4 (26.7)	14 (16.5)	
41,000-60,000	18 (18)	1 (6.7)	17 (20)	
>61,000	35 (35)	7 (46.7)	28 (32.9)	
Living situation: living alone	23 (23)	2 (14.3)	21(24.7)	0.006**
Living with spouse	30 (30)	10 (71.4)	20 (23.5)	
Living with children	20 (20)	0 (0)	20 (23.5)	
Living with spouse and children	9 (9)	2 (14.3)	7 (8.2)	
Living with extended family	14 (14)	0(0)	14 (16.5)	
Living in an institutional home/destitute	3 (3)	0 (0)	3 (3.5)	
Source of income: work	42 (42)	8(53.3)	34 (40.5)	0.353
Children	57 (57)	7(46.7)	50 (59.5)	
Smoking status: never smoked	78 (78)	11(73.3)	67 (78.8)	0.736
Past smoker	22 (22)	4(26.7)	18 (21.2)	
Alcohol intake: never drank	74 (74)	5 (33.3)	69 (81.2)	<0.0001**
Took alcohol before	21 (21)	10(66.7)	11 (12.9)	
Current takes alcohol	3 (3)	0 (0)	3 (3.5)	
Stopped	2 (2)	0 (0)	2 (2.4)	
Supplement use: yes	52 (52)	8 (69.2)	43 (52.4)	0.258
No	43 (43)	6 (30.8)	39 (47.6)	
Teeth problem: yes	42 (42)	8(57.1)	34 (42.5)	0.309
No	52 (52)	6(42.9)	46 (57.5)	
Adult teeth loss: yes	45 (45)	7(46.7)	38 (46.3)	0.981
No	52 (52)	8(53.3)	44 (53.7)	
Dysphagia: yes	2 (2)	1(6.7)	1 (1.2)	0.279
No	98 (98)	14(93.3)	84 (98.8)	
Difficulty in smelling: yes	6 (6)	1(6.7)	5 (5.9)	1.000
No	94 (94)	14(93.3)	80 (94.1)	

**Table 2:** the anthropometry profile and modified mini-nutritional assessment of the participants

Anthropometry profile	All	Male	Female	P-value
Weight	73.7 ±16.5	69.8 ±10.8	74.4 ±17.2	0.322
Height	161.4 ±7.97	172.1 ±6.2	159.5 ±6.7	<0.0001**
BMI	28.7±7.4	23.7±4.1	29.6±7.6	0.004**
Waist circumference	99.9 ±14.97	95.6 ±11.97	100.6 ±15.3	0.253
Hip circumference	111.7 ±14.9	101.6 ±9.2	113.4 ±15.1	0.006**
Waist; hip ratio	0.90± 0.09			0.058
Mid arm circumference	32.9 ±6.3	29.5 ±2.8	33.4 ±6.6	0.03**
Calf circumference	35.8 ±4.98	33.1 ±2.3	36.2 ±5.2	0.03**
Knee height	16.2 ±13.5	15.2 ±2.2	16.3 ±14.5	0.769
Demi-span measurement	77.7 ±6.6	85.4 ±6.8	76.5 ±5.7	<0.0001**
**Modified mini nutritional assessment**				
0-7 (malnourished)	0	0	0	0.754
8-11 (at risk of malnutrition)	27(27.3)	3(20.0)	24(28.6)	
12-14 (normal nutritional status)	72(72.7)	12(80.0)	60(71.4)	
	**60-69 years**	**70-79 years**	**80+ years**	
0-7 (malnourished)	0	0	0	0.339
8-11 (at risk of malnutrition)	17(23.1)	14(30.4)	3(23.1)	
12-14 (normal nutritional status)	42(76.9)	32(69.6)	10(76.9)	

BMI: body mass index

Regarding malnutrition prevalence, the MNA revealed that 27.2% of participants were at risk of malnutrition, while 72.7% had normal nutrition. Among those at risk, females were more affected (88.9%) compared to males. The finding of normal nutrition was predominant among the male gender, those aged 60-69 years, and those above 80 years based on a modified mini nutritional assessment (Table 2).

**The burden of micronutrient deficiencies:** the cost of insufficient micronutrients: serum vitamin A, vitamin D, vitamin B12, and iron levels displaying the profile of participants by gender and age distribution (Table 3). Of all the micronutrients, vitamin A had the highest incidence (10%), followed by vitamin D (1%) which was common in those aged 70-79 (4%). Iron and B12 deficiency seem to be uncommon among individuals. The mean score for males with vitamin A insufficiency was greater than the female counterpart's (194.7 ± 232.7). A comparable circumstance is observed in those between the ages of 60 and 69, whose mean score is higher at 167.0 ± 139.3 than it is in those above 69 (Table 3).

**Table 3:** the profile of participants across gender and age distribution using serum vitamin A, vitamin D, vitamin B12, and iron levels

	Vitamin A			Vitamin D			Vitamin B12			Iron	P-value		Any deficiencies (%)	P value
	**Mean± SD**	**P-value**	**Def N (%)**	**Mean± SD**	**P-value**	**N (%)**	**Mean± SD**	**P-value**	**N (%)**	**Mean± SD**	**P-value**	**N (%)**		
All	162.7±161.1		10(10.0)	124.0±105.9		1(1.00)	1001.1±426.2		0	13.9 ±13.9		0		
**Gender**					**0.758**			**0.360**			**0.299**			**0.875**
Male	194.7±232.7	0.407	2(2.0)	131.8±117.4		0	908.1±328.4		0	14.9 ±4.5		0	2(18.2)	
Female	157.0±146.0		8(8.0)	122.6±104.5		1(1.0)	1018.4±441.5		0	13.7± 3.8		0	9(81.8)	
**Age category**					**0.574**			**0.510**			**0.602**			**0.273**
60-69 years	167.0± 139.3	0.537	3(3.0)	135.6± 119.1		1(1.0)	983.4 ±433.3		0	13.8± 4.0		0	4(36.4)	
70-79 years	166.0±189.8		4(4.0)	115.8 ±92.4		0	1028.0± 437.8		0	13.8 ±3.8		0	4(36.4)	
>80 years	144.0± 127.4		3(3.0)	120.2± 116.1		0	965.2 ±407.5		0	15.0± 4.0		0	3(27.3)	

Def: deficiencies

## Discussion

The present study revealed that none of the participants had malnutrition with 72.7% having a normal nutritional status while 27.3% were at risk of malnutrition. However, a small percentage had micro-nutrient deficiencies with a prevalence of 10% and 1% for vitamins A and D respectively. These deficiencies occurred more among the female gender and participants within the age range of 70-79 years.

Our finding of a prevalent normal nutritional status is at variance with reports from previous studies on nutrition among older adults where a high prevalence of malnutrition was reported [12-17]. The finding of a prevalent normal nutritional status could be attributed to the living arrangement of this cohort. We observed that the majority were living with their spouses, children, and relatives. The quality of relationships with family members and loved ones has some impact on older adult´s dietary patterns influencing them positively [18]. Those who live with family members could have their diets monitored, encouraging a healthy eating lifestyle compared to the elderly living alone or residing in elderly homes [19,20]. Furthermore, marital status could have played some role in the nutritional status observed in our participants; as a majority of our cohorts are married. Studies have shown that older adults whose spouses are alive and are living together tend to eat healthier than those who are widowed [18,21-23]. As individuals age with their spouses, there exists more social interaction and deeper connection which influences feeding habits as healthier meals are prepared and eaten together [24]. However, few studies reported no association between malnutrition and living together [25,26]. Determinants of this contrasting result have not been efficiently expounded, however, it may possibly be due to tooth loss [16,27-29].

Another possible explanation for the prevalent nutritional status could be our participants´ level of education and income level. Most of the participants in our study are literate and are likely to know what nutrients are necessary for their health. Factors such as level of education and income level have been reported to be positively associated with malnutrition [30-33]. In a cross-sectional study by Timpini *et al*. carried out among community-dwelling elderly in Poland, the risk for malnutrition and the effect of different socioeconomic status (SES) indicators were assessed and they found that low educational status was associated with malnutrition [17]. They also indicated that education exposes one to health information that provides adequate dietary knowledge and it guides attitudes and behaviours toward healthy eating and living [17]. With the level of literacy in our cohort, they are likely to be knowledgeable enough to feed on the right meals which play an important role in their nutritional status.

In the same vein, income level has been demonstrated to be independently associated with malnutrition [23]. As consumption is directly related to income status [16], health practitioners have highly recommended dietary intake of proteins and vitamins for older adults [19,34,35]. However, these diets are quite expensive with such being more readily accessible to people with higher financial status [4]. Interestingly, many of our participants, although retired, still receive their pension and most of them stay with their family members who are responsible for their feeding.

Another plausible reason for the prevalence of normal nutritional status among our cohort is the lower prevalence of chronic medical conditions. The absence of medical illnesses promotes functional independence. This could play out in terms of participants being able to make choices for their diets as well as enjoy their meals due to the absence of painful experiences from morbidity and adverse drug reactions from the use of medications [16,36].

Dysphagia is a condition that could lead to malnutrition as a result of difficulty in swallowing resulting in a lower intake of calories and other nutrients [28,29]. However, we observed a very low prevalence of dysphagia among this cohort. A smooth intake of food makes eating enjoyable and thereby increases the consumption of macro-nutrients needed for good health [37]. Although, there was no clear indication that alcohol intake and smoking had a significant influence on malnutrition, however, heavy alcohol consumption has been reported to have a negative effect on nutrition as it impairs the functionality of the liver which deters the smooth metabolism of protein and calories [16,38,39]. Interestingly, alcohol intake and smoking were also non-prevalent in our study with a large percentage of the participants who had never drank nor smoked. This could attest to the low malnutrition status found among this cohort.

Our findings also showed that the participants aged 60-69 years were more likely to have better nutritional assessment than those between the ages of 70-79 years in this study. This is in line with findings from previous studies which indicated that as people age, the risk of malnutrition increases [16,38,39]. Other studies have posited that aging is characterized by changes in body composition, food absorption, and insensitivity to taste which weakens the desire for food [16,29,40]. Moreover, as oral health and dentition deteriorate with aging [27,28,41] food intake and nutritional status of some older adults are affected [16,29]. These could be possible explanations for the lower nutritional status among our participants within the ages of 70-79 years as 55.3% of our participants had tooth problems and tooth loss. As people age, tooth loss is a problem because the dentition deteriorates [16,29]. A previous study on dentition status, malnutrition, and mortality among older service housing residents conducted by Saarela *et al*. found a high prevalence of malnutrition among their cohorts which was attributed to poor dentition with difficulty eating [42]. Their study also reported a lower intake of energy and micro-nutrients in comparison with dentate people [42]. The loss of a tooth can affect chewing and intake of nutrients especially when either the molars or premolars are lost [29]. These posterior teeth are needed for grinding of food particles. The inability to chew food makes eating less enjoyable, this affects the quantity of calories and protein intake which is expected to cause malnutrition [29].

Interestingly, we observed that the males have better nutritional assessment compared to the females. There are possible explanations for this: which could be due to differences that exist in their biological and metabolic processes [22,31,32]. Recently, researchers have indicated that females decompose more lipids and fewer carbohydrates and amino acids compared to males [43]. This decreases the energy requirement and essential nutrients the body needs. In tandem with this, a cohort study among older patients in a hospital in Northern Taiwan reported that gender was associated with nutritional status as the female subjects were slightly worse than of males. They observed that there were differences between the genders in terms of food preference and intake [40,44]. They also noted metabolism differences, and demographic and psychological predictors such as age, education, income, and symptoms of depression as possible reasons for their results.

Individuals may have an adequate level of protein and calories yet have less of micro-nutrients which may not be noticeable. Hence, micro-nutrient deficiency is called hidden hunger. In our study, we found out that our cohorts had normal nutritional status. However, we found a deficiency of vitamins A (10%) and D (1%) among them.

Vitamin A deficiency could be due to inadequate intake of vegetables and fruits containing retinol [44,45], and excessive cooking of foods that contain vitamin A. Fruits such as orange, tangerine, pineapple, and mango are good sources of vitamin A. An insufficient consumption of these fruits may cause a vitamin A deficit. Among our cohorts, the fruits may have not been regularly and adequately consumed like vitamin B12 and serum iron.

This study also indicated a very low prevalence of vitamin D deficiency among the cohorts. As decreased exposure to sunlight and conversion of vitamin D to its active form can cause deficiency [30,46]; the low predominance of vitamin D among the cohorts can be explained by the high exposure to sunlight in this region. The sub-Saharan region has been reported to have a high density of sunlight and low vitamin deficiency [47] compared to a study on other regions of the world located at the lower latitude, such as the Middle East, where they found a high prevalence of vitamin D deficiency, ranging from 50 to 97% [48]. More so, our findings show that the majority of our participants consume natural foods like fish and also use supplements which could have made up for the low vitamin D deficiency [49].

Another explanation for our results is gender differences in food choices [44]. Backing up this preposition, recent research indicated that women had more preference for cakes, full-cream milk, yogurt, butter, apples and pears, bananas, and beverages other than tea and coffee. Men had a preference for eggs, certain meat products including sausage, sugar, and fermented drinks like beer [44]. They found out that women had a significantly higher intake of foods known to be vitamin C-rich, than men. Women also had higher plasma levels of cholesterol, phosphate, and copper, but lower indices of iron and vitamin D status, than men based on the kinds of foods consumed [44].

A study conducted in Britain among old people living on the mainland reported that women between the ages of 65 and 79 years had higher intakes of fat, retinol, vitamin C, calcium, and vitamin E than men but lower intakes of protein, zinc, iron and vitamin D than men [44]. They concluded that differences in the nutritional status of the genders could be explained by the choices of diet [34]. Notably, there were no deficiencies in vitamin B12 and serum iron among our participants. This indicates that the participants take enough fruit and vegetables. Also, a possible explanation could be the daily usage of vitamins and mineral supplements. These supplements increase the immunity of older adults as they contain the necessary micro-nutrients needed to prevent illnesses and other deficiencies [8]. These supplements, as they imply, make up for any occurrence of a low level of nutrients consumed from foods.

**Limitation of study:** we did only the level of vitamins A, B12, D, and serum iron level to determine the burden of hidden hunger.

## Conclusion

Interestingly, our findings showed malnutrition was not apparent among this cohort drawn from a Low- and Middle-Income Country (LMIC). A large proportion had normal nutritional status although some were at risk of malnutrition and a small proportion were deficient of micronutrients; vitamins A and D. The findings present a preliminary knowledge of the nutritional status of older adults in Nigeria; a topic with dearth of information in sub-Saharan Africa and LMICs in general. There is still a need for more comprehensive multisite studies that are generalizable.

### What is known about this topic

- *Micronutrient deficiencies, including vitamins and minerals, are often observed in elderly populations in LMICs due to poor diet and limited availability of fortified foods;*
- *Vitamin A deficiency is common among the elderly in Low- and Middle-Income Countries including Nigeria*.

### What this study adds

- *Notable gender differences in nutritional status of the elderly in this setting;*
- *Elderly females are at higher risk of malnutrition;*
- *Micronutrient deficiencies, particularly in vitamins, are still prevalent, emphasizing the need for targeted nutritional interventions*.
